# Cell-cell interactions that drive tumorigenesis in *Drosophila*

**DOI:** 10.1080/19336934.2022.2148828

**Published:** 2022-11-22

**Authors:** Masato Enomoto, Tatsushi Igaki

**Affiliations:** Laboratory of Genetics, Graduate School of Biostudies, Kyoto University, Yoshida-Konoecho, Kyoto, Japan

**Keywords:** Cell-cell interaction, Tumorigenesis, Tumour progression, Tumour microenvironment, *Drosophila*

## Abstract

Cell-cell interactions within tumour microenvironment play crucial roles in tumorigenesis. Genetic mosaic techniques available in *Drosophila* have provided a powerful platform to study the basic principles of tumour growth and progression via cell-cell communications. This led to the identification of oncogenic cell-cell interactions triggered by endocytic dysregulation, mitochondrial dysfunction, cell polarity defects, or Src activation in *Drosophila* imaginal epithelia. Such oncogenic cooperations can be caused by interactions among epithelial cells, mesenchymal cells, and immune cells. Moreover, microenvironmental factors such as nutrients, local tissue structures, and endogenous growth signalling activities critically affect tumorigenesis. Dissecting various types of oncogenic cell-cell interactions at the single-cell level in *Drosophila* will greatly increase our understanding of how tumours progress in living animals.

## Introduction

Studies in cancer biology have documented that tumour progression is driven by the accumulation of genetic alterations such as activation of oncogenes and inactivation of tumour-suppressor genes. For instance, colorectal cancer is developed by the sequential acquisition of genetic mutations in the *apc, KRas, smad2/4*, and *p53* genes [1]. This indicates that cells clonally develop into malignant tumours, namely ‘clonal evolution’ of tumour cells [2]. However, recent genomic analyses of cancers have revealed that cancer tissues exhibit genetic heterogeneity [3]. Such studies have provided a concept that distinct subclones of tumour cells drive cancer progression via cell-cell interactions [4]. Recent studies in mouse models have shown that clonal diversity is indeed beneficial for cancer development [5–7].

Genetic studies in *Drosophila* have identified crucial tumour-suppressor genes including components of the Hippo pathway and dissected the underlying mechanisms [8]. Particularly, the genetic mosaic technique in *Drosophila* enables to visualize and genetically manipulate cell clones *in vivo* [9,10], which has dissected the molecular mechanisms by which tumours progress towards malignancy [8]. In *Drosophila* imaginal epithelium, clones of cells expressing a constitutively activated form of Ras (Ras^V12^) form benign tumours, which develop into malignant tumours when additional mutations in the apico-basal polarity gene such as *scribble* (*scrib*) or *discs large* (*dlg*) are introduced [11,12]. Ras^V12^ clones with loss of cell polarity cause unlimited growth, invasion, metastasis, and the animal lethality [11,12]. Thus, *Drosophila* is a useful model organism to investigate clonal behaviour of cells with oncogenic alterations *in vivo*. Another achievement of the fly works using the genetic mosaic technique is that it has unveiled the existence of oncogenic alterations that promote non-autonomous tumour progression in surrounding cells via cell-cell interactions [13]. Recent studies in *Drosophila* have shown that oncogenic cell clones drive tumour progression via cell-cell communications with normal epithelial cells, tumour cells with distinct oncogenic alterations, and other type of cells. In this review, we summarize the mechanisms of tumour progression driven by cell-cell communications found in *Drosophila* and discuss the roles of such oncogenic cell-cell interactions in other biological contexts, as well as the similarity between *Drosophila* and mammalian cancer progressions.

## Tumour progression by short-range cell-cell interactions

**Figure 1.**
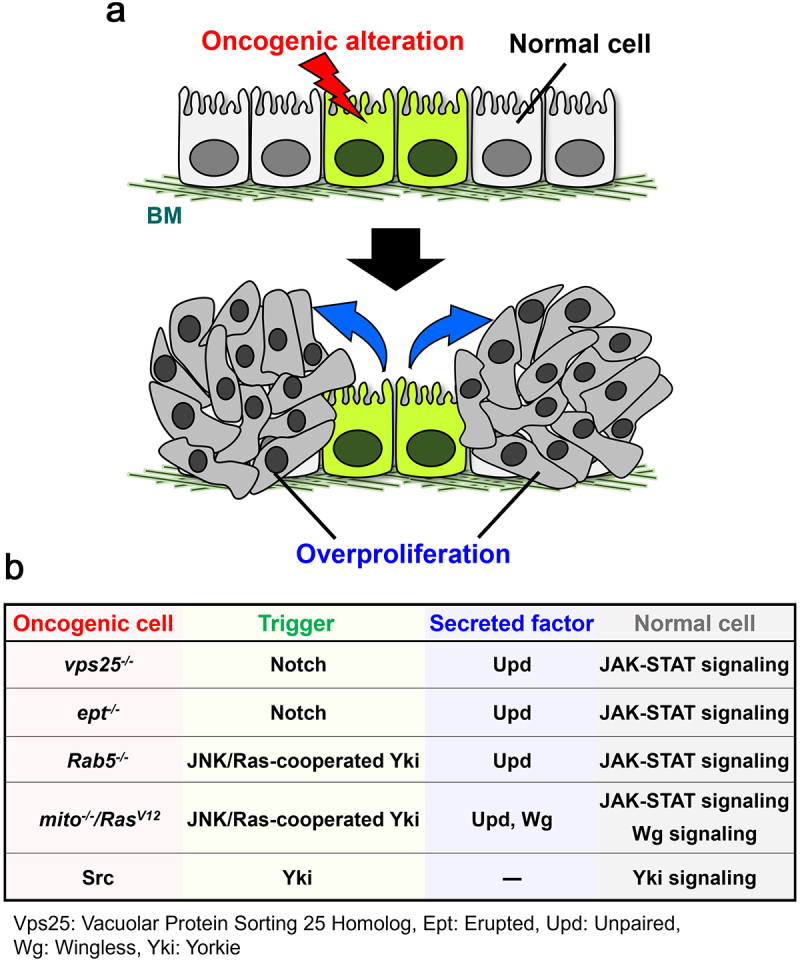
Non-autonomous overgrowth by oncogenic cells in *Drosophila* epithelium. **(a)** Cells with oncogenic alterations (*e.g*. endocytic dysregulation, mitochondrial dysfunction, Ras activation, or Src activation) can promote proliferation of their surrounding cells. **(b)** Summary of oncogenic cells that cause non-autonomous overgrowth. Oncogenic cells: mutant cells with genetic alterations, BM: Basement membrane. See text for details.

Genetic studies using *Drosophila* imaginal discs have discovered that oncogenic mutations can drive tumour progression via cell-cell communications (Figure 1a). Such mutations include endocytic dysregulation, mitochondrial dysfunction, apico-basal polarity loss, and Src activation. Notably, these mutant cells behave as ‘oncogenic niche cells (ONCs)’ that do not overgrow but instead provide tumour overgrowth and invasion in their neighbours [13]. In this section, we describe the mechanisms by which short-range cell-cell interactions between ONCs or normal cells drive tumour progression.

### Endocytic dysregulation

Genetic screens in *Drosophila* have shown that clones of cells mutant for endosomal sorting complex components, *vps25*, or *erupted* (*ept*, a *tsg101* homolog) in the imaginal disc cause overproliferation of surrounding cells [14–17]. These mutations cause endosomal accumulation of Notch, which activates Notch signalling. This leads to the induction of its target Unpaired (Upd, an IL-6 homolog), which causes non-autonomous overgrowth of surrounding tissue by activating JAK-STAT signalling [14–17] (Figure 1b). Likewise, mutant clones for *Rab5* (an early endosome component) drive non-autonomous overgrowth of surrounding tissue via Upd, but the underlying mechanism is different from that caused by *vps25* or *ept* mutants. Clones of *Rab5* mutant cells activate EGFR-Ras and Eiger (a TNF homolog)-JNK signalling, which cooperate to activate the transcriptional coactivator Yorkie (Yki, a YAP/TAZ homolog) via inactivation of the Hippo pathway, leading to the induction of its target Upd [18] (Figure 1b). Although the mechanism of Upd induction is different between *vps25* and *Rab5* mutants, both mutant cells exhibit the same cell cycle status. *Rab5* mutant cells enter the endocycle by downregulating Cyclin B via cooperation between JNK and Yki-DIAP1 (*Drosophila* inhibitor of apoptosis protein 1) signalling, thereby becoming polyploid giant cells [19]. Similarly, mutant clones of *vps25* or *avalanche* (*avl*, a *syntaxin7* homolog) show polyploidization phenotype, which is caused by Eiger/TNF-JNK signalling [19]. Although it is not yet clearly understood how JNK and Yki-DIAP1 downregulate Cyclin B in endocytic mutant cells, these observations suggest that endocytic mutants commonly cause endoreplication via cooperation between JNK and Yki. Importantly, cooperation between JNK and Yki not only drives tumour progression but contributes to tissue homeostasis. Upon epidermal injury, cells around the injury site cause polyploidization via cooperation between JNK and Yki, and the polyploid giant cells seal the space lost by tissue damage [20–22]. In addition, in a mouse model of Fuchs endothelial corneal dystrophy, polyploid cells compensate the space lost by cell death to ensure tissue homeostasis in corneal endothelium that shows increased cell death with age [20]. Thus, cell polyploidy could play crucial roles in both tumour progression and tissue repair.

### Mitochondrial dysfunction

**Figure 2.**
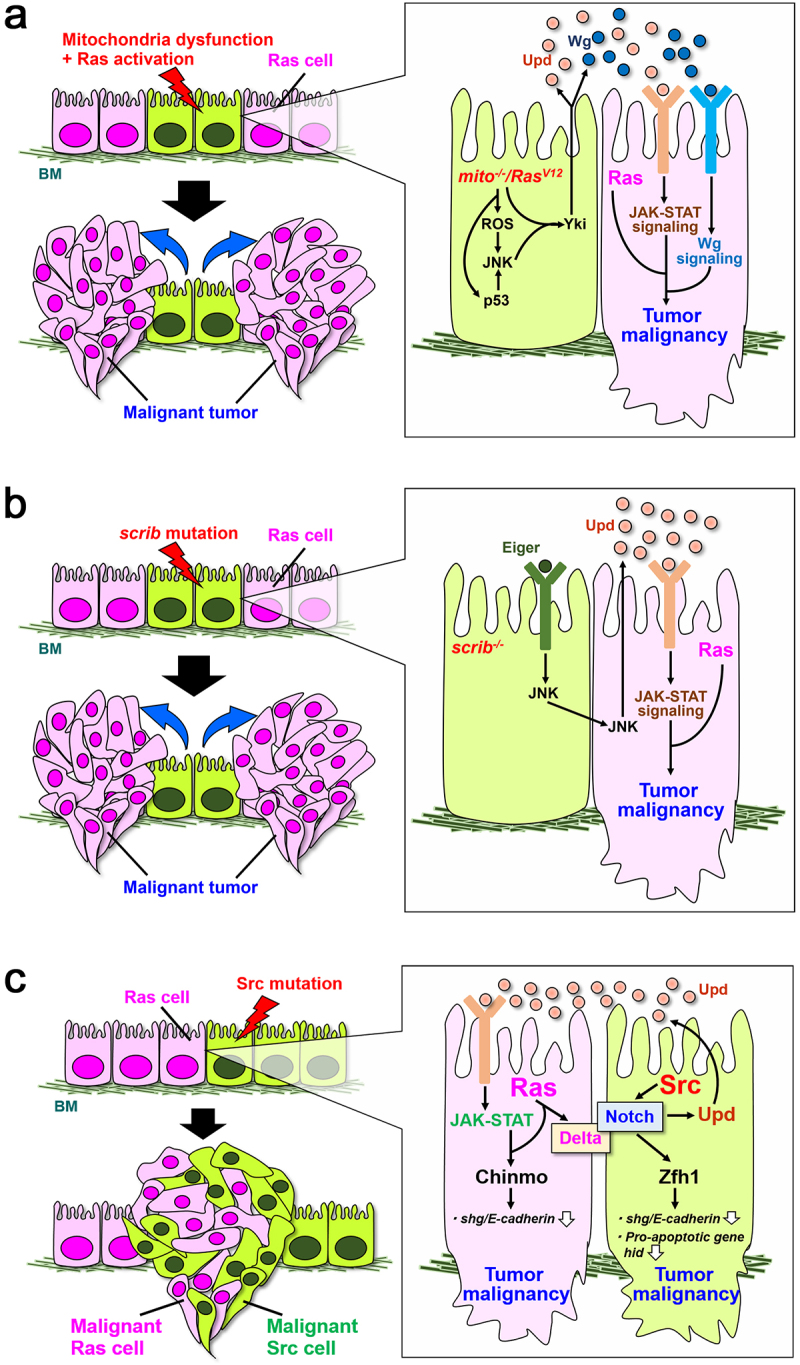
Malignant transformation of benign tumours via cell-cell interactions in *Drosophila*. **(a)** *mito^−/−^/Ras^V12^* cells activate JNK signalling via production of ROS and activation of p53. Cooperation between JNK and Ras signalling activates Yki, which causes invasion of neighboring Ras cells through induction of Upd and Wg. **(b)** *scrib* mutant cells activate Eiger-dependent JNK signalling, which propagates to neighboring Ras cells. JNK signalling in Ras^V12^ cells induces Upd, which causes invasion via JAK-STAT signalling. **(c)** Delta from Ras cells activates Notch in Src cells, and elevated Notch signalling causes invasion of Src cells via Zfh1-mediated downregulation of *shg* and *hid*. At the same time, Notch signalling in Src cells induces Upd, which activates JAK-STAT signalling in adjacent Ras cells. Activated STAT in Ras cells represses *shg* expression via Chinmo to cause invasion of Ras cells. BM: Basement membrane.

Mutational activation of *Ras* oncogene is found in many cancers [23,24]. A genetic screen using Ras^V12^-expressing clones in *Drosophila* imaginal epithelium has identified a series of mutations in the components of the mitochondrial respiratory complex as inducers of non-autonomous overgrowth of surrounding tissue [25]. Mechanistically, Ras^V12^ clones with defects in the mitochondrial respiratory complex (hereafter referred to as *mito^−/−^/Ras^V12^*) overproduce reactive oxygen species (ROS), which activates JNK signalling. JNK and Ras signalling cooperatively activate Yki via inactivation of the Hippo pathway, leading to overproliferation of surrounding cells via induction of Upd and Wingless (Wg, a Wnt homolog) [25] (Figure 1a,b). A constitutively activated form of JNK kinase (Hemipterous) causes non-autonomous overgrowth when cooperated with Raf (a downstream effector of Ras) in the eye imaginal disc [26], suggesting that Ras signalling cooperates with JNK signalling via Raf for Yki activation. Notably, *mito^−/−^/Ras^V12^* cells cause tumour malignancy in their neighbouring Ras^V12^ benign tumours [25] (Figure 2a). Intriguingly, *mito^−/−^/Ras^V12^* cells undergo cellular senescence via enhanced activation of JNK signalling through cooperation between ROS production and p53-mediated cell cycle arrest and thus exhibit SASP (senescence-associated secretory phenotype) [27]. SASP is a phenomenon that senescent cells highly express secreted growth factors, inflammatory cytokines, chemokines, and proteases [28,29]. These SASP factors induce cell proliferation, invasion, metastasis, chemoresistance, and immune suppression in neighbouring cells, causing non-autonomous cancer progression [28,29]. Related to this, after ionizing radiation (IR) irradiation of the imaginal disc bearing Ras^V12^ clones, Ras^V12^ cells with higher p53 expression support survival of neighbouring Ras^V12^ cells with lower p53 expression by inducing Upd [30]. Similar to *Drosophila mito^−/−^/Ras^V12^* cells, IR irradiation in human lung cancer cells causes production of mitochondrial ROS and G2/M phase arrest [31]. Notably, it has been shown in human cell lines and mice that mitochondrial dysfunction causes cellular senescence that triggers a SASP-like phenomenon called MiDAS (mitochondrial dysfunction-associated senescence), which induces HMGB1, TNF-α, and IL-10^32^. It has been shown that low cellular NAD^+^/NADH ratio and AMPK-induced p53 activation are required for the induction of MiDAS [32]. The fact that somatic mutations in mitochondrial DNA (which encode the components of the mitochondrial respiratory complex) are frequently found in cancers [33] suggests a scenario that Ras-activated cells with mitochondrial dysfunction drive cancer development and recurrence through SASP/MiDAS factors via cell-cell interactions.

### Cell polarity defects

Apico-basal polarity is essential for maintaining epithelial integrity and its disruption is often a critical event for cancer progression [34]. Epithelial tissue entirely mutant for an apico-basal polarity gene *scrib* or *dlg* massively overgrows and develops into invasive tumours [35]. Interestingly, however, clones of polarity-deficient cells surrounded by wild-type cells are eliminated from epithelial tissues by cell death [11,12], a phenomenon celled cell competition that is driven by short-range cell-cell interaction [36–39]. Eiger-JNK signalling plays central roles in the elimination of polarity-deficient cells by promoting cell death [40,41] and cell extrusion [42], and is also activated in neighbouring wild-type cells to induce engulfment of polarity-deficient cells [43]. While Eiger-JNK signalling contributes to elimination of polarity-deficient cells, it drives tumorigenesis in these cells when Ras signalling is simultaneously activated [44,45]. JNK and Ras signalling cooperate to inactivate the Hippo pathway via intracellular F-actin accumulation, thereby causing tumour overgrowth [46]. Interestingly, JNK signalling in *scrib* clones drives tumour progression when Ras signalling is activated in their neighbouring cells [47] (Figure 2b). In this case, JNK signalling in *scrib* clones propagates to neighbouring Ras^V12^ cells, thereby causing metastatic overgrowth of Ras-activated clones via activation of JAK-STAT signalling triggered by JNK-dependent Upd induction [47] (Figure 2b). In *scrib*-induced cell competition, it has been shown that JNK-induced *upd* expression in neighbouring wild-type cells promotes cell proliferation and thus compensates for the lost space after *scrib* cell elimination [47,48]. In mammalian MDCK cell cultures (Madin-Darby canine kidney cells/Dog kidney epithelial cells), it has been shown that cells with simultaneous Ras^V12^ overexpression and *scrib* knockdown (*scrib-KD*) overproduce mitochondrial ROS, which elevates TOR signalling in these cells and causes engulfment of neighbouring *scrib-KD* cells [49]. In mammals, Ras-activated cells surrounded by normal cells are excluded from the epithelial sheet of MDCK cells or mouse intestinal epithelia [50,51]. On the other hand, similar to *Drosophila, scrib-KD* MDCK cells are excluded from epithelial cell sheet, although it is not caused by JNK but by p38 MAPK signalling [52,53]. Nevertheless, *scrib-KD*/*Ras^V12^* MDCK cells outcompete neighbouring *scrib-KD* MDCK cells by entosis, suggesting that oncogenic mutant cells can clonally expand their territory by cell competition in the early stage of carcinogenesis.

### Src activation

The tyrosine kinase Src is a classical oncoprotein and its expression and activity have been shown to be correlated with cancer development [54]. In *Drosophila* imaginal epithelium, cells activating Src (a c-Src homolog) are eliminated by cell competition when surrounded by wild-type cells [55,56]. Elimination of Src-activated cells when surrounded by wild-type cells is also observed in mammalian cell lines and zebrafish embryo [57,58]. Given that Src activity and expression are increased in cancers[54], Src-activated cells may evade cell competition during cancer progression. Interestingly, it has been shown that Src cells transform into malignant tumours when Ras is activated in neighbouring cells in *Drosophila* imaginal disc [59] (Figure 2c). Ras^V12^ and Src clones increase the cell surface ligand Delta and its receptor Notch, respectively, and thus Delta-Notch interaction occurs at the boundary between these clones. Activated Notch signalling in Src clones induces the transcriptional repressor Zfh1 (a ZEB1 homolog), which transforms Src cells into malignant tumours by downregulating *shotgun* (*shg, an E-cadherin* homolog) and a pro-apoptotic gene *hid* [59] (Figure 2c). Simultaneously, Notch signalling in Src cells induces Upd, which activates JAK-STAT signalling in neighbouring Ras^V12^ cells. STAT activation in Ras^V12^ cells causes Chinmo-mediated repression of *shg* and thus induces tumour malignancy [59] (Figure 2c). It has recently been shown that the ETS family transcriptional factor Pointed (Pnt, a ETS1 homolog) induces cellular senescence downstream of Ras signalling [60], and therefore Ras^V12^ clones show limited tumour growth. Notably, loss of cell polarity in Ras^V12^ clones causes Yki-mediated induction of microRNA *bantam*, which cancels Ras-induced cellular senescence by downregulating Pnt [60]. In the process of tumour progression driven by interaction between Ras and Src clones, it is still unclear how Ras^V12^ cells evade Pnt-mediated cellular senescence. One possibility is that Src cells induce non-autonomous activation of Yki in neighbouring Ras^V12^ cells. Indeed, while Src clones surrounded by wild-type cells are eliminated by JNK-dependent cell death, Src cells simultaneously propagate Yki activity to neighbouring cells in a JNK-dependent manner [56] (Figure 1a,b). Notably, heterogeneity of Ras and Src clones in the tissue causes interdependent tumour malignancy, whereas clones activating both Ras and Src just overgrow but do not show invasive behaviour.

## Tumour regulation by nutrient signalling

**Figure 3.**
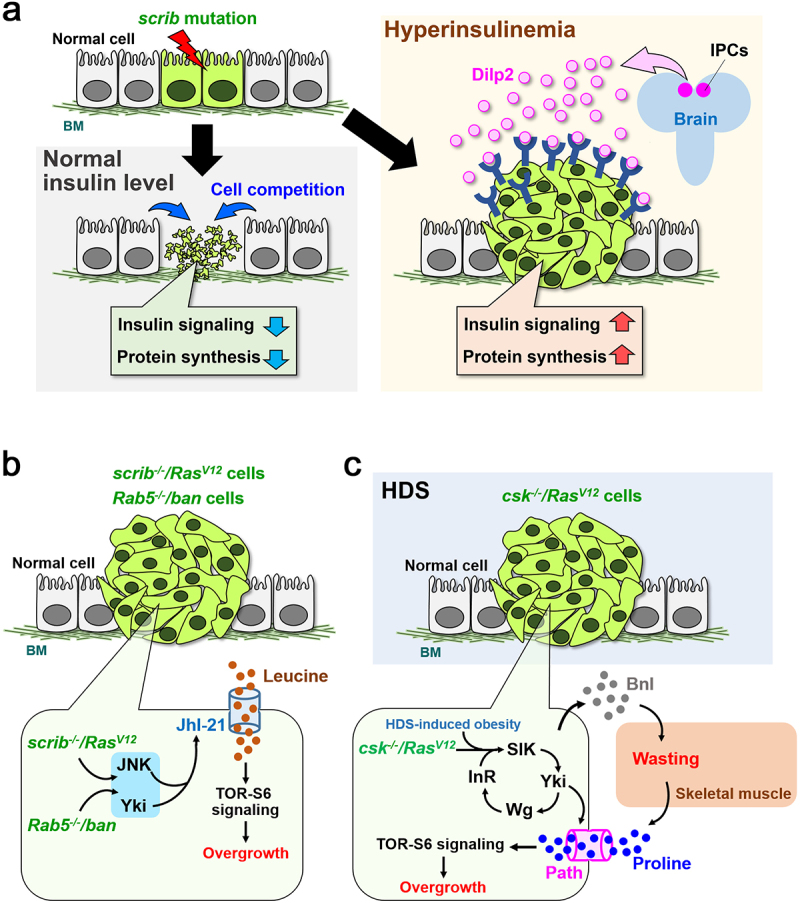
Nutrient signalling that drives tumour progression. **(a)** At normal insulin level, *scrib*^−/−^ cells are eliminated by cell competition when surrounded by wild-type cells. Under hyperinsulinemia, IPCs in the brain overproduce Dilp2, which activates insulin signalling in *scrib*^−/−^ cells. Upon insulin signalling activation, *scrib*^−/−^ cells acquire high protein synthesis levels and initiate tumorigenic overgrowth. **(b)** *scrib*^−/−^*/Ras^V12^* or *Rab5*^−/−^*/ban* clones activate JNK and Yki, which cooperatively upregulate the amino acid transporter JhI-21. Increased JhI-21 promotes uptake of leucine, causing tumour overgrowth via TOR-S6 signalling activation. **(c)** In animals fed with HDS, *csk*^−/−^*/Ras^V12^* clones produce Bnl, which systemically causes skeletal muscle wasting to release proline into hemolymph. Circulated proline is selectively incorporated via the amino acid transporter Path into *csk*^−/−^*/Ras^V12^* clones and causes tumour overgrowth via TOR-S6 signalling activation. BM: Basement membrane.

Recent transcriptomics and metabolomics analyses have revealed that human cancer cells utilize nutrients for cell proliferation, survival, invasion, and metastasis [61]. Interestingly, oncogenic cells that cause non-autonomous tumour progression in *Drosophila* are also dependent on nutrient signals. Clones of polarity-deficient cells are eliminated by cell competition from imaginal epithelia, while this elimination is abrogated by hyperinsulinemia [62]. Briefly, in mutant flies heterozygous for *chico* (the insulin receptor substrate, IRS1-4 homolog), *scrib* clones evade cell competition and develop into tumours by elevated insulin signalling [62] (Figure 3a). Mechanistically, insulin-producing cells (IPCs) with reduced *chico* levels overproduce *Drosophila* Insulin-like peptide 2 (Dilp2), which causes hyperinsulinemia and remotely activates insulin-TOR signalling in *scrib* mutant clones, thereby evading cell competition by increasing intracellular protein synthesis [62] (Figure 3a).

It has also been shown that amino acid metabolism plays pivotal roles in tumorigenesis in *Drosophila* imaginal epithelium. For instance, *scrib^−/−^/Ras^V12^* clones alter mitochondrial respiratory activity and thus produce ROS to induce autophagy in neighbouring cells. Elevated autophagy in neighbours locally supplies amino acids to *scrib^−/−^/Ras^V12^* tumours, which assists tumour overgrowth [63]. Intriguingly, *scrib^−/−^/Ras^V12^* clones in the eye discs also induce autophagy in other organs such as fat bodies and muscles, which causes organ wasting, leading to a release of amino acids/sugars into circulation that would promote tumour growth [63,64]. These data suggest that *scrib^−/−^/Ras^V12^* clones actively take amino acids from neighbours to promote tumour growth. A recent study identified an amino acid essential for tumour growth of *scrib^−/−^/Ras^V12^* clones. In *scrib^−/−^/Ras^V12^* clones, JNK and Yki cooperate to upregulate the amino acid transporter Juvenile hormone Inducible-21 (JhI-21, an L-amino acid transporter 1 LAT1 homolog), which activates TOR-S6 signalling by uptaking leucine to promote tumour growth [65] (Figure 3b). JhI-21 is also upregulated by cooperation between JNK and Yki in invasive tumours caused by *Rab5* mutation with overexpression of microRNA *bantam* (*Rab5^−/−^*/*ban*) [65]. Thus, similar to mammalian cancer [66], leucine uptake is essential for tumour progression of *scrib^−/−^/Ras^V12^* and *Rab5^−/−^*/*ban* clones in *Drosophila*.

Src-activating cells require other amino acids for tumorigenesis. In flies fed with high dietary sucrose (HDS), Ras^V12^ clones with mutations in *C-terminal Src kinase* (*csk*, a negative regulator of Src) (*csk^−/−^/Ras^V12^*) induce Branchless (Bnl, an FGF homolog) that causes systemic muscle wasting, leading to increased circulating amino acids (Figure 3c). *csk^−/−^/Ras^V12^* clones then uptake proline via the SLC36 transporter Pathetic (Path) that is upregulated by Yki activation, thereby promoting tumorigenesis via TOR-S6 signalling activation [67] (Figure 3c). In this case, JhI-21 has little effect on tumour growth of *csk^−/−^/Ras^V12^* clones [67]. On the other hand, cells with elevated Src42 (one of fly Src proteins) require methionine-mediated TOR activation for cell proliferation in the wing disc [68]. Thus, oncogenic cells may selectively uptake favourable amino acids by regulating expression of specific transporters for their proliferation, survival, invasion, and metastasis. TOR activation could be a common feature of the nutrient signalling in oncogenic cells. TOR signalling is known to enhance ribosome biosynthesis [69], and interestingly, imbalanced protein synthesis levels between cells could be a critical factor for triggering cell competition [62,70–72]. These observations imply that oncogenic cells acquire higher ribosomal biogenesis via nutrient-dependent TOR activation, thereby transforming into winners of cell competition.

Given the crucial role of TOR signalling in tumours, TOR could be an ideal target for anti-cancer therapies. However, there is a big problem that TOR inhibition would also affect viability of healthy cells [73]. Blockage of amino acid transporters may overcome this problem. In fact, human JhI-21 homolog LAT1 is upregulated in many tumour tissues [74,75] and preclinical studies have shown that pharmacological inhibition of LAT1 is effective to suppress cancer growth [74,75]. In particular, the LAT1 inhibitor JPH203 is currently evaluated in clinical trials for biliary tract cancers [74]. Consistently, the LAT1 inhibitors (BCH and KYT0353) possess tumour-suppressive activity against *scrib^−/−^/*Ras^V12^ tumours in *Drosophila* [65]. However, the LAT1 inhibitor has little effect on tumour growth of *Rab5^−/−^/ban* clones [65]. Interestingly, transcriptome analyses followed by *Drosophila* genetics identified the TMEM135-like gene *CG31157* that attenuates the effect of the LAT1 inhibitor in *Rab5^−/−^/ban* tumours. Removing *CG31157* in *Rab5^−/−^/ban* clones allowed the LAT1 inhibitors to suppress tumour growth [65]. Thus, *Drosophila* can be a powerful model to identify molecules involved in nutrient metabolism and drug resistance in tumorigenesis.

## Tumour progression by microenvironmental factors

Interactions of oncogenic mutant cells with microenvironmental cells such as stromal cells, immune cells, and endothelial cells also play important roles in tumour progression [76]. Recent studies in *Drosophila* have shown oncogenic interactions between mutant epithelial cells and microenvironmental factors during tumour progression.

### Mesenchymal cells

**Figure 4.**
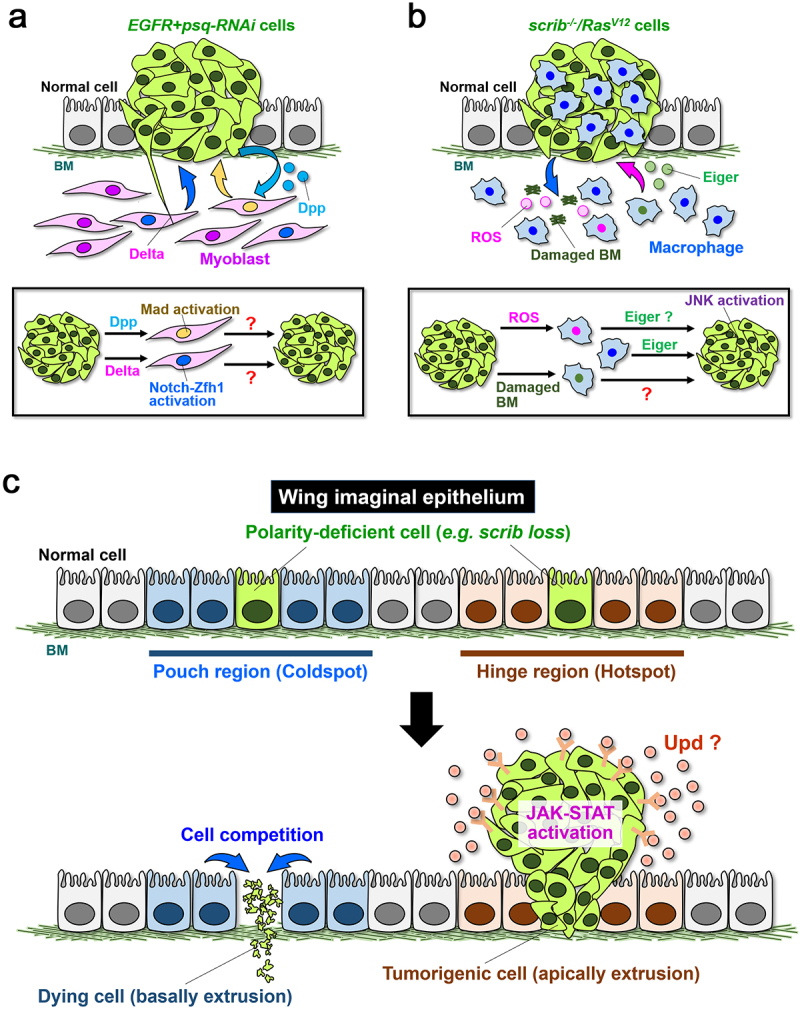
Tumour progression via interaction between tumour cells and microenvironmental factors. **(a)** In the wing imaginal disc, *EGFR+psq-RNAi* cells secrete Dpp, which activates Mad in myoblasts. The Mad activation promotes tumour growth of epithelial *EGFR+psq-RNAi* cells. *EGFR+psq-RNAi* cells also activate Notch signalling in myoblasts through Delta provided by cytoneme and cause overproliferation via Notch-mediated Zfh1 induction. **(b)** *scrib*^−/−^*/Ras^V12^* tumours generate ROS and damaged BM, which recruit macrophages. Macrophages secrete Eiger, which activates JNK signalling in *scrib*^−/−^*/Ras^V12^* clones to promote tumour growth. **(c)** In the wing disc, polarity-deficient cells in the pouch region (Coldspot) undergo apoptosis and are basally extruded. Conversely, those cells in the hinge region (Hotspot) apically delaminate and cause tumourigenic overgrowth via activation of JAK-STAT signalling in the lumen. BM: Basement membrane.

In the wing imaginal disc, EGFR-activated cells with loss of *pipsqueak* (*psq*), a transcription factor involved in epigenetic control, massively overgrow [77]. In this process, *EGFR+psq-RNAi* cells produce Decapentaplegic (Dpp, a TGF-β/BMP homolog), which activates Mad (a Smad homolog) in myoblasts (progenitor cells for flight muscles) that exist at the basal side in the notum region of the wing disc (Figure 4a). The Mad activation increases the number of myoblasts, which in turn promotes proliferation of epithelial *EGFR+psq-RNAi* cells [77] (Figure 4a). It has been also been shown that *EGFR+psq-RNAi* cells activate Notch in myoblasts via its receptor Delta provided by a long membrane protrusion called cytoneme [78]. Activated Notch signalling in myoblasts upregulates Zfh1, which is essential for overproliferation of *EGFR+psq-RNAi* cells [78] (Figure 4a). In this reciprocal communication between tumour cells and stroma cells, it is still unclear how Mad and Zfh1 in myoblasts promote epithelial tumour growth. Two independent studies using single-cell transcriptomics analysis have shown genetic heterogeneity in myoblast populations during normal development [79,80]. Notably, epithelial cells control the number and location of myoblasts via secretion of two FGF family ligands (Thisbe and Pyramus) and change the transcriptional programme in myoblasts by inducing the ligand Hedgehog [80]. Thus, two transcriptional regulators Mad and Zfh1 may generate genetic diversity of myoblasts that promote epithelial tumorigenesis. Similar to the observations in *Drosophila*, an RNA-sequencing analysis using Head/Neck cancer patients-derived xenografts has shown that TGF-β-induced gene expression is upregulated in stromal cells, which increases the cancer-associated fibroblasts (CAFs) marker α-smooth muscle actin (α-SMA) [81]. Jagged (a ligand for Notch) from epithelial tumours has been shown to interact with Notch2 in fibroblasts, thereby inducing CAF phenotype in ductal breast carcinoma [82]. In addition, it has been reported that ZEB1 is increased in stromal cells of human breast cancers and *ZEB1* deletion suppresses mammary tumour formation in a mouse model of breast cancer [83]. Notably, CAFs comprise of diverse subpopulations in human cancers such as breast, head/neck, lung, and pancreas cancers [84]. Thus, studies on tumour-stroma interactions in *Drosophila* could contribute to understand human cancer development.

### Immune cells

Macrophages penetrate tumour tissues and support tumour progression by regulating tumour growth, invasion, metastasis, angiogenesis, and immunosuppression [85]. It has been shown that *Drosophila* macrophage-like cells plasmatocytes (hereafter macrophages) are recruited to malignant tumours in the imaginal epithelium. *scrib^−/−^/Ras*^V12^ clones generate damaged basement membrane and reactive oxygen species (ROS), which are required for macrophage recruitment [86–88]. It has been shown that macrophages in tumour tissues produce Eiger, which promotes growth of *scrib^−/−^/Ras*^V12^ clones via JNK activation [45,86] (Figure 4b). In contrast, genetic ablation of macrophages promotes overgrowth of imaginal discs entirely mutant for *scrib* [88]. In addition, the number of circulating macrophages is increased by PVF1 (a PDGF and VEGF homolog) derived from *dlg* mutant tumours, which causes tumour cell death by inducing Eiger [89]. In this process, macrophages produce the Toll ligand Spätzle, which acts in the fat body to promote secretion of Eiger via Toll signalling [89]. The fat body also induces antimicrobial peptide Defensin, which causes cell death of *dlg* mutant tumours [90]. On the other hand, allograft experiments using larval tumours have shown that tumour mass of *scrib-RNAi+Ras^V12^* cells is unaffected by loss of macrophage [91].

Thus, the role of fly macrophages in tumorigenesis is still obscure. A possible explanation for this is that different macrophage subsets infiltrate into different tumours. In mammals, there are two subtypes of macrophages, M1 (pro-inflammatory) and M2 (anti-inflammatory) macrophages, and their roles and functions are plastically changed in response to tissue environmental cues [92]. Intriguingly, single-cell transcriptome analyses using *Drosophila* larvae have revealed that fly macrophages are also diverse cell populations [93–96] and its clusters are dynamically changed in different physiological and pathological conditions, such as septic injury [95], bacterial infection, and parasitic infection [94–96]. Another possible explanation is that fly macrophages also engage in the adaptive immunity-like response. In *Drosophila* embryo, macrophages remove apoptotic debris via the engulfment receptor Draper that is upregulated by calcium-induced JNK signalling, and this phagocytosis primes inflammatory response whereby macrophages rapidly respond against the secondary bacterial infection [97]. In adult flies, macrophages incorporate viral double-stranded RNA from virus-infected cells and synthesize virus-derived complementary DNA (vDNA) by endogenous reverse transcriptases [98]. This vDNA biogenesis allows *de novo* synthesis of viral secondary siRNA (vsRNA), which is secreted from macrophages by exosome-like vesicles for systemic immunity [98]. Thus, fly macrophages seem to have aspects of both innate and adaptive-like immunities. It is well known in mammals that the adaptive immune system efficiently attacks cancer cells [99]. If fly macrophages exert anti-tumour activity similar to mammalian cytotoxic T lymphoma and B cells, it might be a cause of the complexity of tumour-associated macrophages act in imaginal epithelium.

### Local tissue microenvironment

Epithelial tissue structures differ even within the same tissue, and its patterning is thought to be a crucial factor for tumour initiation and development. For instance, colon cancers frequently develop at the rectum and sigmoid colon regions [100]. Similarly, mammary carcinogenesis often occurs in the upper outer quadrant of breast [101,102]. However, it is still elusive why cancers originate from specific regions of the tissue. Studies in *Drosophila* have elegantly dissected the origin of tumorigenesis and regeneration in the wing imaginal disc. In the pouch region of the wing disc, clones of *scrib* cells are basally eliminated from the tissue, whereas those cells induced in the hinge region delaminate from the apical side and initiate tumorigenesis in the lumen [103]. Genetic analysis has shown that cells in the hinge region endogenously possess high levels of STAT activity as compared to the other regions and show basally enriched microtubules[103]. Such tissue-intrinsic cytoarchitecture generates ‘tumor hotspot’ that initiates tumorigenesis. Hinge cells in the wing disc also show resistance to IR- and drug-inducible cell death via elevation of the STAT effector Zfh2 and Wg signalling [104,105]. IR-induced caspase activation provides stem cell-like properties in hinge cells by increased ribosome biogenesis for tissue regeneration [105,106]. These observations suggest that hinge cells are potentially cancer-stem cells (CSCs) that are involved in tumour initiation and recurrence. Notably, in a mouse model, the JAK-STAT pathway is activated in subpopulations of recurrent tumours that comprise polyclones after tumour regression [107]. In addition, blocking JAK-STAT signalling using anti-IL-6 antibody (siltuximab) and a STAT3 inhibitor (LLL12) suppresses colony formation of stem-like cells derived from prostate cancer patients [108]. Notably, in *Drosophila*, apically delaminated polarity-deficient cells originate tumorigenesis, while oncogenic cells that are basally delaminated can cause non-autonomous tumour growth. In the wing disc, the basally delaminated cells with chromosomal instability (CIN) (*e.g. bub3- or rod*-depleted cells expressing a caspase inhibitor p35) cause epithelial tumorigenesis via JNK-mediated Wg expression [109]. Delaminating cells with CIN generate dysfunctional mitochondria, which produce ROS to cause JNK-dependent cellular senescence [110]. Alongside *mito^−/−^/Ras^V12^*, CIN also causes cellular senescence through mitochondrial dysfunction and triggers non-autonomous growth. Thus, downregulation of mitochondrial function may be an etiology of cancer development through oncogenic cell-cell interactions.

## Conclusions and Perspectives

**Figure 5.**
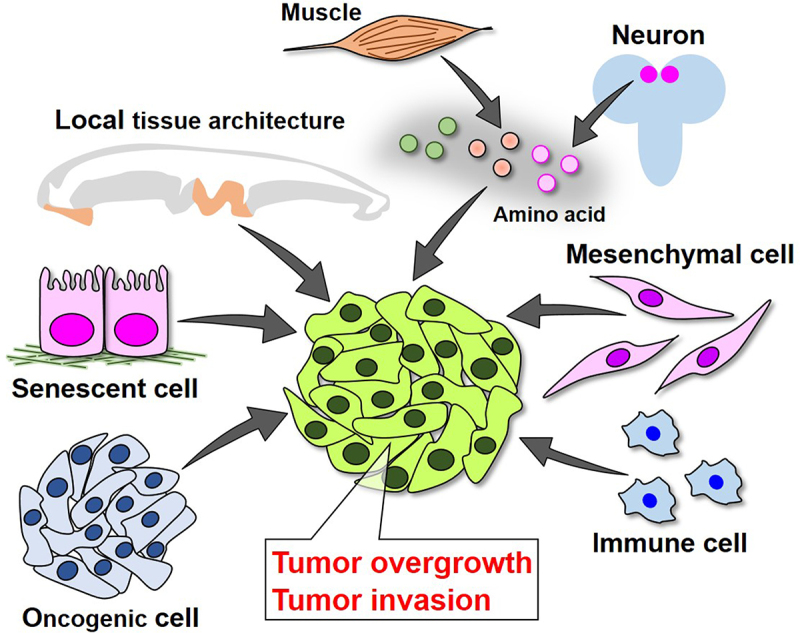
Cell-cell interactions driving cancer progression in *Drosophila* epithelium.

Genetic studies in *Drosophila* have provided a brilliant platform to understand the basic principle of tumour progression via cell-cell communications. In this review, we summarized the studies on tumour progression driven by local and systemic cell-cell interactions in *Drosophila* (Figure 5). Recent transcriptomics analyses have revealed that diverse cells with distinct gene expression profiles emerge in imaginal epithelia with *scrib* mutations during tumour development [111,112]. A similar genetic heterogeneity has been shown in tumorigenic follicle cell populations activating Notch in *Drosophila* ovary [113]. Thus, diverse cells exist in the same tissue with tumours, raising an interesting possibility that divergent clonal evolution occurs during the process of tumour progression in *Drosophila*. Given that recent multi-omics techniques allow us to analyse cells in human cancer tissues at the single-cell level and that single-cell techniques and applications using *Drosophila* tissues/organs are rapidly developing [114], dissecting cell-cell interactions within *Drosophila* tumours at the single-cell level will greatly increase our understanding of the mechanisms of tumour initiation and progression.
